# A qualitative analysis of a culturally adapted PCIT training for black and latine clinicians: creating communities for providers of autistic youth

**DOI:** 10.3389/frcha.2025.1517169

**Published:** 2025-06-03

**Authors:** Harlee Onovbiona, Felipa Chavez, Lauren Quetsch, Ashley Scudder

**Affiliations:** ^1^ Department of Psychological Science, University of Arkansas, Fayetteville, AR, United States; ^2^ Department of Psychology, Florida Institute of Technology, Melbourne, FL, United States; ^3^ Department of Human Development & Family Studies, Iowa State University, Ames, IA, United States

**Keywords:** community, training, adaptation, culture, Parent Child Interaction Therapy (PCIT)

## Abstract

Parent-Child Interaction Therapy (PCIT) is a widely known evidenced-based treatment (EBT) that has been used with Latine, Black, and neurodiverse children to improve the parent-child relationship and reduce challenging behaviors. Although considerable efforts have been made to disseminate PCIT to the wider community, fewer strides have been made to reach Black and Latine families in underserved communities—especially for families with neurodiverse children. One method to bridge the service gap for Black and Latine families is to train Black and Latine clinicians who primarily serve Black and Latine communities. Thus, the current pilot utilized a qualitative design and the Ecological Validity Model to examine the clinical and cultural impact of a culturally infused PCIT training pilot, the Creating Communities Initiative, for Black and Latine mental health providers (*N* = 8)., Black and Latine clinicians highlighted several beneficial cultural adaptations (e.g., racially-ethnically matched community) and barriers (e.g., low caseloads) to training completion and competency development. Overall, the results of the culturally infused PCIT training pilot provide a useful template for future dissemination efforts of PCIT to culturally diverse providers and families.

## Introduction

More than ever before, research and clinical attention is being directed toward improving mental health practices' inclusivity and cultural responsivity. Efforts include developing cultural adaptations to evidence-based treatments (EBTs), discussing treatment barriers to traditional outpatient services, and implementing interventions in underserved communities (1–3). Despite such efforts, disparities persist in accessing culturally informed EBTs, especially among families with intersectional, marginalized identities (4). Culturally informed EBTs addressing the specific needs and values of a cultural community are desperately needed; they can promote psychological familial growth within the cultural environments in which Latine and Black families reside and create welcoming, empowering, and meaningful therapeutic spaces for families across cultural and neurotype presentations.

In particular, Black and Latine families often face mental health disparities, some of which have been attributed to poor access to services, discrimination, and poor culturally responsive care (3–5). Furthermore, Black and Latine families with an autistic child often face further marginalization due to stigma from their peers, lack of neurodiverse care in their communities, and misdiagnoses by culturally uninformed providers stemming from the intersection of these marginalized identities (6, 7). For example, Latine caregivers of autistic youth report language barriers with professionals, hindering access to linguistically congruent treatment and resources in their native language (8, 9). Re-experiencing racism within discriminatory healthcare systems also play an important role in accessing quality care for Black and Latine families (2, 6, 10), as they can increase families' exposure to bio-psycho-social vulnerabilities and racism-informed societal conditions (11). Findings demonstrate that a significant proportion of families of Black or multiracial autistic youth experience racism and discrimination in seeking care for their child (22%) and that cultural differences between themselves and their providers negatively influence their progress [16.3%; (12)]. Taken together, care that is delivered without considering the values and lived experiences of Black and Latine families and that are rooted in racism, may increase the likelihood of mistrust towards treatment providers who are believed to be aligned with discriminatory healthcare systems (12, 13). In fact, in a mixed-method study examining perspectives from Black families of autistic youth on the diagnostic and treatment process, 75% of families indicated that the professionals they worked with did not display cultural humility, patience, or understanding (6). Thus, fostering culturally-informed treatments that align with the values of Black and Latine families, along with provider trust, are especially paramount for achieving effective interventions when working with Black and Latine families of autistic youth (14), which is significantly hampered by a predominantly white mental health workforce.

While research demonstrates that many white providers endorse wanting to help communities of color, uncertainty and discomfort may limit their willingness to broach racial and ethnic discussions and applications in treatment (15). To date, Black and Latine clinicians make up only 9% of the psychology workforce (16, 17), but even fewer are trained in EBTs and feel competent in providing care to autistic individuals (18, 19). Trainings for EBTs have remained *white spaces* (17), which generally refers to “geographic areas perceived … to have boundaries and protections for white privilege; white people; and their behaviors, culture, and norms” (20). The perpetuation of white spaces in trainings has been maintained by organizational, structural, and systemic factors that promote a Eurocentric model and precludes the inclusion and representation of diverse individuals in EBT training spaces. Accordingly, both researchers and families of color call for greater representation of Black and Latine mental health professionals (6, 7). In a previous study, one Black mother of an autistic child shared: “*I would just say try to hire more people of color. Like, if that was, like an initiative or something, just trying to get more, because it would be nice for us, you know, a client to see their therapist, or at least more therapists in the building that look like them*” (6)*.* Increased cultural competence as well as cultural and linguistical similarities support Black and Latine families feel better understood and comfortable when seeking mental health care (6, 21). One strategy for integrating EBTs within the cultural context of Black and Latine communities lies in training Black and Latine clinicians who primarily serve Black and Latine families and are competent in providing care to families of autistic youth (22). As such, in a parallel process, EBT training models should take place in culturally responsive environments including cultural adaptations that are tailored toward Black, Latine, and autistic populations.

## Framework: the ecological validity model

The *Ecological Validity Model* (EVM) was proposed by Bernal and colleagues (23) in an effort to provide a culturally adaptive treatment guide for working with Latine clientele. Accordingly, in an area where culturally adaptive training models are lacking, the EVM provides a useful template for designing culturally adaptive EBT models. The EVM recommends that adaptations fit the cultural needs of the desired population across eight dimensions: *Language, Persons, Metaphors, Content, Concepts, Goals, Methods*, and *Context* (23, 24). Researchers dedicated to enhancing trainings for Black and Latine mental health providers should also consider the impact of these dimensions relevant to the training, the trainer/s, the trainee/s, as well as the clients and families of color they serve.

The first dimension, *Language*, includes the use of linguistically concordant methods, resources, and supports for trainees and their families. The importance of this element has been highlighted across the literature by families, researchers, and clinicians as a priority for Spanish-speaking families to receive quality care (24, 25). Similarly, among a sample of Black families of autistic youth, nearly 80% of caregivers indicated not understanding the language used to communicate with them (26). Indeed, prior studies have also found that language adaptations are associated with greater intervention acceptance and retention rates (27).

Next, the dimension of *Persons* refers to the important role that cultural identity (e.g., gender, race, ethnicity) plays for those involved the intervention and the training. Specifically, this dimension refers to the ethnic/racial identity matching among the clinician, treatment resources, and the client in the EBT implementation space as well as the identity matching among the trainer(s), training resource materials, and the trainees in an EBT training space. In a previous study assessing the experiences of Black caregivers of autistic youth navigating the service delivery system, 15% of caregivers expressed wanting more access to Black professionals and providers (6). Similarly, Black therapists have expressed desires to have safe workplaces with other Black therapists with whom to share their experiences (28).

*Metaphors* include the use of symbols, sayings, and idioms throughout an intervention or training to create an inclusive environment and to explain concepts in a way that are culturally understandable. Some examples include the incorporation of *Dichos* (i.e., sayings, folklores, storytelling, or urban legends) that signify the valued strength and empowerment of Black people. The latter is seen in the legend of *John Henry*, a Black man whose strength out produced the work of machines in the advent of the industrial revolution, or the *superwoman* archetype for Black women that embodies their androgenous roles to be everything to everyone in the uplifting of their families. Such metaphors within the respective cultures serve as bridges to conveying and understanding aspects of the training/intervention. While *Content* is comprised of elements of a(n) training/intervention that integrate the teaching of cultural information and knowledge (e.g., Black and Latine values, autism knowledge, historical experiences unique to each group), *Concept* refers to the overall alignment of the intervention or training model with the values and needs of the trainees and families (strength, empowerment, self-reliance, community). Unfortunately, cultural factors are underwhelmingly considered in many master's level training programs—with these programs producing large numbers of community mental health professionals (28). According to Black and Brown families of autistic youth, autism knowledge is also underwhelmingly prevalent in the psychology and medical professional community, as one mother even recommended that professionals receive additional autism training: *“I don’t know how doctors get training after they get their Ph.D., I know they go to conferences and stuff like that, so maybe more opportunities for them to learn about autism. Cause they just don’t know a lot for them to be in the middle to be dealing with as many autistic children as they do*” (6). The dimension of *Goals* emphasizes the need to clearly communicate goals of the training/intervention to trainees and families. It further suggests that the goals of the training should align with the trainee and families of color's goals and values (e.g., notions of collectivism, *familismo—importance of family, fictive and non-fictive kinship supportive networks*).

The next EVM element includes *Methods*, which are culturally adapted and non-adapted practices and activities included in a(n) training/intervention to help families or trainees reach their goals such as implementing methods that reduce barriers and increase accessibility. Trainings may include attempts to increase its accessibility using virtual platforms. Trainings may also employ methods of social interchange and engagement that help build rapport and connection among the trainer and trainees through common experiences (known as *personalismo*). Lastly, *Context* encompasses the socio-cultural components (e.g., acculturative stress, economic conditions, career stage, socio-cultural events, and agency support) that impact the trainees and the families they are serving. Ultimately, context largely influences the trainee's ability to implement an intervention to clients in the real-world setting. The consideration of context is critical, and when it is overlooked, this may lead to decreased engagement, efficacy, acceptability, and satisfaction (6). In fact, a finding from Onovbiona and colleagues (6) examining the influence of barriers on treatment engagement and satisfaction among Black families of autistic youth, highlighted that families who experienced more practical or contextual barriers, were more likely to report decreased satisfaction and effectiveness ratings for the treatments they engaged in. Concentrating training efforts for Black and Latine providers—which utilize these approaches and dimensions—is desperately needed. Studying these efforts may then provide justification for the reconceptualization and reorganization of EBT trainings—overhauling mental health systems that fail to prioritize the needs of Black, Latine, and autistic communities.

On average, 50% of children with ASD exhibit disruptive behaviors (78). While medications are the most common method used to treat disruptive behavior in youth with ASD, they are also associated with significant side-effects, and often diminish in effectiveness when discontinued (79, 80). Intensive behavioral interventions focusing on the child, such as applied behavioral analysis, have demonstrated success in reducing problem behavior (81), but they can be time-intensive, and specialized providers are unavailable in many under resourced communities (82, 83). Treatment efforts for disruptive behaviors have recognized that parental skill development is essential for generalization and family well-being in ASD populations (84); however, behavioral interventions in typical, community-based settings are often implemented with low intensity and limited parent involvement, thus demonstrating the need for more therapist training in proven practices in order to ensure high-quality treatment implementation (41). Nationwide research has indicated that clinicians of color are vastly outnumbered by white clinicians; in 2013, 5.1% of psychologists and 7–17.5% master's level clinicians were people of color. These differences are especially pronounced in specialty providers who serve autistic youth [i.e., (7)]. This disparity likely reflects systemic barriers in accessing advanced mental health training. Therefore, therapist training for Black and Latinx providers in evidence-based treatments for youth with ASD and disruptive behaviors is critical to addressing gaps in diagnostic clarification, service provision, and utilization. One evidence-based program which has shown substantial effectiveness with Black and Latinx families in community setting is Parent-Child Interaction Therapy (PCIT, 29, 85, 86). In (*redacted for review*), 480 kids have received PCIT services, 73% of these children are Black and 10% are Hispanic. There are 7 designated provider organizations with a total of 31 PCIT clinicians actively treating children, however less than 1% of clinicians identify as part of the Black or LatinX community. Furthermore, no clinicians have received in-depth specialized training in providing PCIT to children and families with ASD although it has demonstrated comparable effectiveness in reducing disruptive behaviors of children with ASD. This proposed initiative aims to reduce the disparity gap by training Black and Latinx community-based providers to deliver PCIT to underserved Black and Latinx families of children.

## The current study

The present study explored a novel, culturally grounded training in Parent-Child Interaction Therapy (PCIT), a widely disseminated intervention for child disruptive behaviors. A small cohort of Black and Latine mental health providers (and their supervisors) from (*redacted for review*) were recruited to pilot a culturally informed training adaptation for PCIT with autistic youth (known as the *Creating Communities Initiative*). Goals of the training included improving clinician competencies across several domains: Black and Latine cultural competency in PCIT treatment applications, autistic competency, and PCIT competency. Clinicians and supervisors were interviewed at the end of the training year to determine (a) strengths of the training impacting clinician competencies, (b) training barriers limiting clinician competencies, and (c) suggestions for training improvements with future cohorts of clinicians from historically marginalized backgrounds.

## Method

### EBT: parent-child interaction therapy

PCIT is an evidenced-based behavioral parent training program developed to reduce challenging behaviors in children ages 2 ½ to 7 years (30). Although PCIT was originally created for and normed on largely neurotypical Eurocentric populations (31), recent adaptations have focused on implementing PCIT in neurodiverse [e.g., (32, 33)] and culturally diverse samples (1, 34). The standard model of PCIT is family centered and trains caregivers in positive parenting strategies across two phases (i.e., Phase 1: *Child Directed Interaction* [CDI], Phase 2: *Parent Directed Interaction* [PDI). In the first phase of treatment (CDI), parents learn how to better connect with their children through developmentally appropriate play-based skills (i.e., the PRIDE skills: *Praise*, *Reflection*, *Imitation*, *Description*, *Enjoyment*) and selective attention for positive child behavior. Caregivers strive for goal criteria of skills prior to moving on to the second phase of treatment. PDI then focuses on increasing child compliance and parental consistency. Throughout PCIT, providers deliver feedback in real time to the caregiver via a Bluetooth device in the caregiver's ear. The provider remains behind a one-way mirror while caregivers engage in play with their child in a playroom. Families graduate from the program when (a) caregivers meet goal criteria for both CDI and PDI, (b) child disruptive behaviors are within typical limits, and (c) caregivers feel confident in their abilities to handle their child's behavior.

To become a Certified PCIT Therapist, professionals must have (a) graduate education (i.e., a master's degree or higher in a mental health field with independent licensure/working towards licensure or be a psychology doctoral student), (b) basic PCIT training (e.g., 40 h/5-day PCIT training, 2-day advanced PDI training), (c) consultation training (e.g., 1 year of twice monthly consultation on cases, must complete at least two cases), and (d) skill review (i.e., treatment sessions must be observed by a certified PCIT Trainer). Procedural fidelity was measured using the standard session integrity checklists provided by the program developers in the PCIT protocol for CDI Skill mastery, PDI Skill mastery, CDI Teach and Coach, and PDI Teach and Coach sessions (pcit.org). Fidelity progress was monitored using Canvas indicating when individual trainees achieved each of the skills during both the 5-day training and the 2-day advance training. The average level of skill acquisition for CDI Skill Mastery was 81% of trainees, while the average for PDI Skill mastery was 49% of trainees. All trainees successfully participated in a CDI teach, CDI coach, PDI teach, and PDI Coach with one to two volunteer families, effectively implementing the PCIT skills relevant for each type of session. In addition, participants were regularly monitored for treatment fidelity in the review of their tracked caseloads and respective weekly Eyberg Child Behavior Inventory (ECBI) and Dyadic Parent Child Interaction Coding System (DPCIS) scores during biweekly consultation calls for informing treatment decisions during the year-long training, as well as additional video case reviews as per the certification requirements indicated by PCIT International Association (PCIT-IA). Accordingly, trainees were trained to 80% reliability in DPCIS coding, while also addressing cultural considerations in DPICS coding to account for variability with the remaining 20% allowance. For more information on PCIT and certification criteria, visit pcit.org.

### Participants

Clinicians working in Medicaid-serving, local behavioral health agencies in (*redacted for review*) were recruited via email flyers. Overall, clinicians across 10 agencies applied for the training. Interested clinicians were invited to participate in the training who met the following inclusionary criteria: (a) identified as Black or Latine and (b) provided direct mental health services to Black and Latine children and families. Clinicians consented prior to enrolling and participating in the study. The study recruited a cohort of 8 Black and Latine clinicians and 4 administrators to participate in the 40 h introductory PCIT training, 2-day advanced training, year-long bi-weekly consultation calls, and video or in-person skill review. A total of 8 clinicians completed the qualitative interviews.

The racial and ethnic breakdown of the providers was as follows: 62.5% Black, 37.5% Latine and bilingual in English and Spanish. Participating providers were mostly cisgender females (75.0%), specialized in clinical psychology (57.1%), and identified with a cognitive-behavioral orientation (42.9%). In total, providers had an average of 4-years of clinical experience (*SD* = 3.41). Approximately half of the providers (52.9%) endorsed prior experience working with clients below the age of 8, and the majority indicated having a higher caseload made up of Black clients (68.6%), with a minority serving Latine clients (31.4%; see Table 1).

**Table 1:** Clinician interview sample demographics*.*

Variable		*M* (SD)	*n* (%)*
Age		31.71 (3.04)	
Years of Clinical Practice		4.43 (3.41)	
Percentage of Experience work with children 8 and younger		52.86 (33.77)	
Percentage of Black clients		68.57 (24.78)	
Percentage of Latine Clients		31.43 (25.45)	
Clinical Experience	Early (<6 years)		5 (62.5%)
	Experienced (>6 years)		3 (37.5%)
Agency Support	Yes		5 (62.5%)
	No		3 (37.5%)
Gender	Female		6 (75.0%)
	Male		2 (25.0%)
Ethnicity	Latine		3 (37.5%)
	Non-Latine		5 (62.5%)
Race	Black		5 (62.5%)
	Latine		3 (37.5%)
Primary Language	English		5 (62.5%)
	Bilingual		3 (37.5%)
Theoretical Orientation	Family Systems		1 (14.3%)
	Cognitive/Behavioral		3 (42.9%)
	Eclectic		2 (28.6%)
	Humanistic		1 (14.3%)
Specialty	Social Work		1 (14.3%)
	Clinical Psychology		4 (57.1%)
	Counseling		1 (14.3%)
	Other (e.g., early intervention)		1 (14.3%)

*n = 8. Eight clinicians completed the interview, but one clinician did not finish the entirety of the demographic survey.

### Procedures

The *Creating Communities Initiative*^1^ was developed through meaningful community partnerships between local organizations (i.e., behavioral health organizations, research foundations) that had the shared goals of (a) increasing access to EBTs for Black and Latine community clinicians and (b) improving care for autistic individuals and underserved communities.

#### Training team

The *Creating Communities Initiative* training and consultation efforts were led by a neurotypical Afro-Latina Ph.D. Clinical Psychologist, with extensive experience in supporting and providing care to Black families in PCIT treatment dissemination and developed the culturally infused curriculum content for the adapted PCIT training, given their 20-year history in multicultural training as clinical graduate faculty. The training was also supported by two white female neurotypical Ph.D. Clinical Psychologists with expertise in PCIT, autism, and dissemination of EBTs to underserved communities. Lastly, the team included two female neurotypical clinical psychology doctoral students (one Black, one Latina) with clinical and leadership experience supporting, garnering perspectives, and advocating for Black autistic and Latine communities.

All members of the training team have demonstrated a commitment to advancing equity, racial justice, and promoting EBPs that support historically marginalized groups. The lead trainer has over 20-years of experience in teaching multicultural course curriculum and trained over 60 graduate students in PCIT as a University within agency trainer, housed within an Autism treatment center. With an accumulation of 18 years implementing PCIT and TCIT at local community centers serving at risk Black and Latine youth and their families, the lead trainer developed a tips sheet offering clinical/training guidelines for the practical applications of PCIT principles in working with Black families in treatment, in support of the Black Lives Matter social movement. The host and regional (secondary) trainer have provided clinical consultation to community based PCIT clinicians for over 10 years, serves on the PCIT International Task Force on Policy and Advocacy, and has exercised a commitment to hosting clinicians and researchers who are interested in racial empowerment. The Clinical Psychology faculty member is dedicated to exploring the intersectionality of neurodiversity and historically marginalized identities and how these impact the rate and timeliness of autism diagnoses, racism and prejudice in autism-based treatments, and stigma of autism in minoritized populations and cultures. The two clinical psychology doctoral students have a combined 7 years' experience of delivering treatment to Black and Latine clients, leading local and national antiracism advocacy efforts, amplifying the voices of the Black and Latine community through research and community endeavors, exploring research topics relevant to historically marginalized communities, and recruiting Latine and Black participant samples.

#### Culturally-informed PCIT training adaptation model

Clinicians participated in a 40 h PCIT introductory training and a 2-day advanced training hosted on a virtual platform. Clinicians were provided with essential binders, handouts, and logins necessary to complete clinical PCIT training competencies. Spanish materials were provided for trainees who served Spanish-speaking families. Clinicians were encouraged to initiate PCIT with at least two families over the training year and participate in 24 bi-weekly consultation calls. At the end of the training year, clinicians were invited to participate in individual semi-structured interviews on their impressions of the training.

The PCIT training model used in the present study was tailored and guided by elements from the EVM, including *Language, Persons, Metaphors, Content, Concepts, Goals, Methods*, and *Context* (35). The training *Method* utilized was a hybrid model, including virtual and in-person training opportunities to promote accessibility and flexibility for the trainees. Within the *Language* dimension, trainees were provided with PCIT protocols, handouts, and materials translated in Spanish to better serve their Spanish-speaking families. In addition, trainees were greeted in Spanish and English at the start of every training session. Discussions centered around the African American Vernacular English (AAVE) were also integrated into the training discussions around coding using the Didactic Parent-Interaction Coding System (36). *Persons* adaptations from the EVM included the racial and ethnic diversity within the leadership of the training team (the training was led by a Afro-Latina clinical psychologist, supported by two white female clinical psychologists, and supported by a Black graduate student and Latina graduate student), inclusion of a African American volunteer family to practice the skills learned in the training, discussions in the training about the intersection for race and disability and how it impacts the experiences of Black and Latine families, as well as the racial and ethnic matching of the trainees and clients. Throughout the training, cultural *Metaphors* were used to connect and engage with the trainees and to further help the trainees employ the information and skills to the Black and Brown families they were serving (e.g., Using African proverbs in the training slides, the “Come to Jesus Sermon” on how to introduce PCIT to Black and Latine clients, and discussing the coercive cycle with a cultural relevant symbol [e.g., *Chancleta*], (36).

In addition to the standard goals included in PCIT trainings, the training *Goals* also aligned with the values of Black and Latine individuals. In particular, goals of creating a community of Black and Latine clinicians in Philadelphia and of improving clinician cultural competencies and competencies in serving autistic youth were established. The *Content* of this training was adapted to include information and discussions centered around the cultural experiences of Black and Latine families. Some examples include discussions and didactics about discrimination trauma and racism, racial-ethnic disparities in diagnoses and treatment, values of Black and Latine families, “Healthy Paranoia” of the medical system (87), and racial and ethnic matching implications (e.g., shared experiences with families). *Concepts* infused within PCIT were also adapted to promote family empowerment (e.g., using PRIDE and PDI skills to empower, support, and promote safety for Black and Brown families).

Lastly, the training team identified socio-cultural components that could impact the trainees, their engagement in the training, as well as the families they were serving. Therefore, *Context*-related adaptions were implemented to increase accessibility and better support the trainees as they worked to serve Black and Latine families within their community. For example, the training was provided at no-cost, ideas were generated on how to make PCIT more accessible for families with fewer resources [e.g., Dollar Tree Bag, Transportation Voucher; (36)], funding for agencies to purchase additional PCIT resources (e.g., manuals, one-way mirror, time out room build out) was provided, and clinicians were encouraged to engage in advocacy and in the community (36, see text footnote 1). Further, during the training year, two national discriminatory social events occurred (e.g., Buffalo Shooting, Ulvade school shooting); time was allocated during the training to reflect and discuss the events and how it potentially impacted the trainees and their clients.

### Semi-structured interviews

Each participant was asked to participate in a qualitative interview at the completion of the training year regardless of if they completed the entirety of the training. Interviews were conducted by one Latina and two Black female doctoral psychology graduate students who were not involved with the implementation of the training. Interviewers were ethnically/racially matched with participants. Participants received a $50 Amazon gift card at the completion of the interview. The average interview duration was 30 min.

Participants were asked about the strengths of the training, challenges and impressions of the training, and cultural components of the training that impacted their experience and competencies (e.g., “*What resources were most helpful during the training?*” “*What are your thoughts on participating in a training conducting by trainers from different racial and ethnic backgrounds (i.e., Black lead trainer, white supportive trainers, graduate student Black and Latina trainers)?*”; see Supplementary Appendix A). Interviews were conducted in English using Zoom and recorded. Each interview was transcribed verbatim.

### Analytic strategy

The interviews were uploaded to QSR N*Vivo 11, a coding software program that aids in theme generation and coding analysis. Given the small cohort size of clinicians enrolled in the pilot training (*N* = 12), the research team interviewed as many clinicians as were willing to consent. A total of eight clinicians agreed to participate in the interviews (*N* = 8; see Table 1). Malterud and colleagues (37) concluded that the sample size of qualitative studies is largely determined by information power, which is guided by the depths of the study aims, the diversity of the participants, theory application, and the richness of the interview dialogue. The final sample size was determined to be adequate given that the study integrated applied theories, contained rich interview dialogue, had narrow study aims, and had highly specific populations (37).

For the coding process, a thematic analysis approach was initially used to generate themes among the interview data (38). One Black graduate-level researcher and one Afro-Latina clinical psychologist individually reviewed the interview transcripts and created preliminary themes from the data set. Multiple coordination meetings were conducted between the two researchers to collaborate, consolidate, and eliminate redundant themes. After utilizing an inductive analysis approach, themes were created for the EVM framework elements (e.g., Language, Persons) and sorted from the inductive analyses into the EVM framework model to reach a final coding scheme. The interviews were then collaboratively coded by the same researchers using the final code scheme as a codebook.

## Results

**Figure 1:**
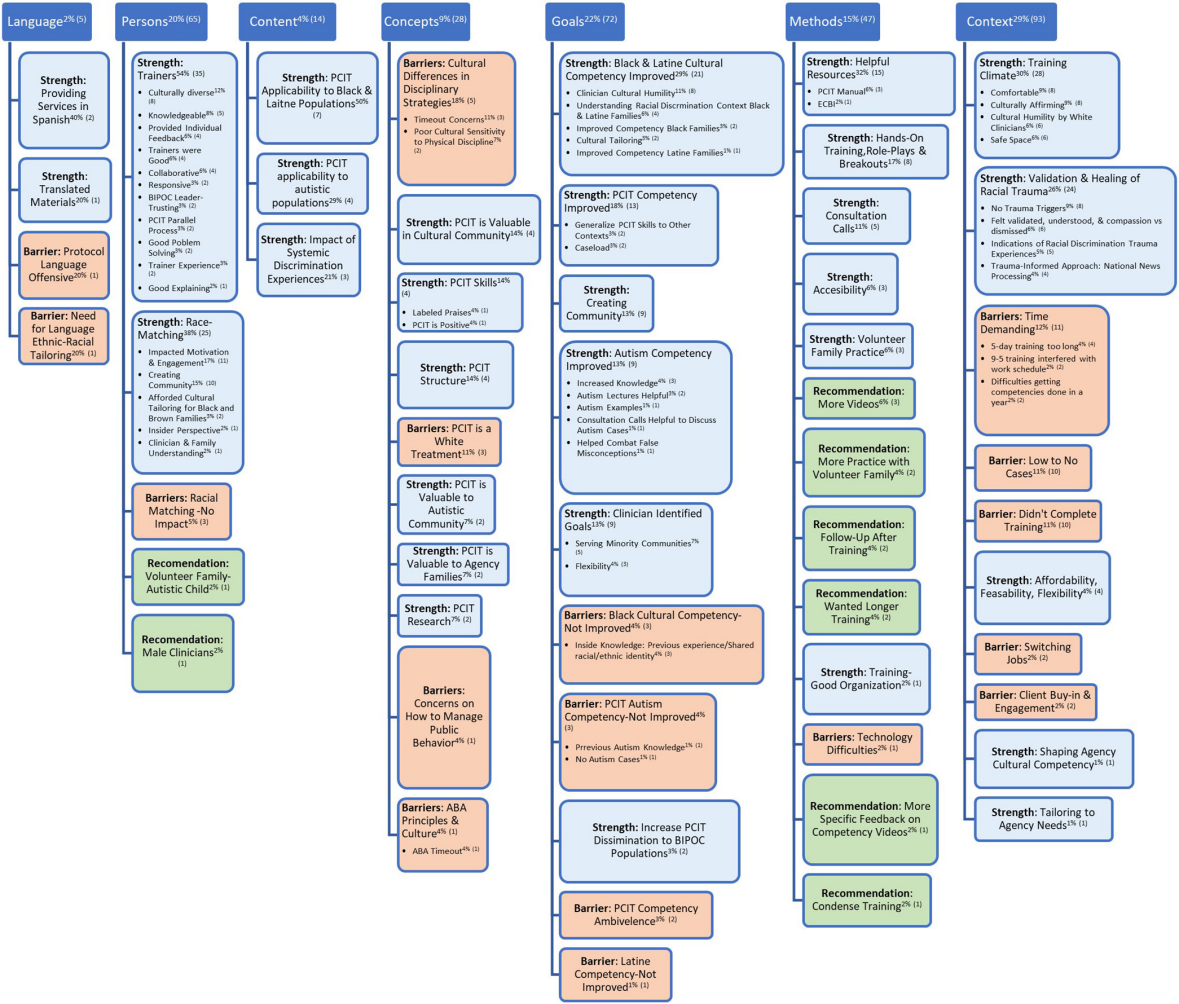
Superscripts refer to the percentages of coding references for a particular theme and the number of coding references for a particular theme in parentheses.

The aims of the study were to determine (a) strengths of the training that may have impacted clinician competency, (b) barriers of the training that may have limited clinician competency, and (c) how this training could be further improved for future cohorts of clinicians from historically marginalized backgrounds. Overall, the following themes and subthemes arose within the EVM dimensions: *Context*, *Goals*, *Persons*, *Methods*, *Concepts*, *Content*, and *Language* (for definitions and list of quotes, see Table 2). Although, incorporated into the culture infused PCIT training curriculum, no themes arose surrounding the *Metaphors* EVM dimension, potentially because they were less salient to the group after the completion of the training year. The proportions of themes that arose differed across strengths, barriers, and recommendations. Figure 1 illustrates the themes and subthemes endorsed by the trainees.

**Table 2:** Example participant quotes broken down by theme.

Theme	Elements
**Language (2%):** Using culturally appropriate language to deliver the training and linguistically relevant resources and supports for trainees to use with their families.	*“The translated material was really helpful when some of the clients that I was speaking to needed to translate that information into Spanish. Although I can speak both languages, sometimes I struggle to find words. Nothing to do with the language. It's just* *…* *easier to have that information already translated for me*”. [Strength-Translated Materials] “*Some parents come [in] shy, maybe because they don't know English, or they believe that it will be English* *…* *and I will say [this happens more] with Hispanic [clients]* *…* *because we live in a community where a lot of people feel shy to speak [English]* *…* *They don't know the language, or they [are afraid they are] going to be made fun of or maybe they're not going to be understood. So being able to have that flexibility* *…* *helps*”. [Strength-Providing Services in Spanish] “*The most challenging, I would say, is the actual protocol itself. I would say a lot of the words in it aren't very culturally inclusive for everyone. So sometimes we found ourselves kind of like trying to find a different word that would fit, you know, our community, because sometimes you know, some words can be offensive to some families, because they may try and say like “Oh are you trying to say that I'm probably harming my child or something?”* [Barrier-Protocol Language Culturally Offensive]
**Persons (20%):** The role of cultural identity (e.g., race, ethnicity, gender) differences and similarities between trainees and trainers as well as trainees and the clients in shaping the training experience and the therapy relationship.	*“I think it's good to have* *…* *individuals from different cultures to implement the training because they may bring different perspectives that we may not be aware of. So, especially with cultural humility, you have to keep yourself in check and keep questioning yourself about other people [‘s] culture. So, if you're exposed to those cultures, you can have a general idea of how to be more culturally competent*”. [Strength-Trainers-Culturally Diverse] “*I work with my.,, Hispanic community and the African American community. So, having clinicians, we don't often like [see]* *…* *we only see each other at big meetings when we go to [CONFERENCE NAME]. So, having a small group of clinicians that are mainly Hispanic and African Americans it made it quite significant. So, it made me, you know, when I read the title, [to] make it a community, you know* *…* *culturally competent, it made it quite inviting and interesting for me*”. [Strength-Persons-Racial-Matching-Creating Community] “*Having a trainer* *…* *also from a similar background* *…* *made me feel like I could do it* vs. *being like, ‘I don't know about this’, but to hear that it could work, and it does work. So that encouraged me to implement the PCIT*”. [Strength-Persons-Racial-Matching-Impacted Motivation and Engagement].
**Content (4%)**: Inclusion of cultural information and knowledge (e.g., Black and Latine values, historical experiences, traditions, customs, unique experiences) into the teaching of the intervention, client conceptualization, and training space.	*“So, we had a day where we kind of talked about discrimination in the Black community and* *…* *our experiences in our own cultures and with the world. And [NAME OF LEAD CLINICIAN], one of the [Afro-Latina lead] trainers, she described the time where her son was actually like stopped by the police and how* *…* *it was, you know, very scary for her* *…* *her Black son being stopped by a cop, and she luckily was there* *…* *and was able to* *…* *kind of like intercept the situation. But we* *…* *know nowadays, sometimes, the parents are not there, and they [the police] don't care. So, it was really nice to be able to kinda still be trying to talk about PCIT, but ultimately kind of talking about the elephant in a room. Where we may be giving our parents this therapy, giving them this training for the better, the family and stuff, but there is still these outside forces in the world who* *…,* *don't care…”* [Strength-Content-PCIT applicability to Black and Latine Families & Impact of Systemic Discrimination Experiences] “*[TRAINER] did a presentation like twice like relating PCIT to autism. So that was really nice to kinda tie that in our agency*”*.* [Strength-Content-PCIT Applicability to Autistic Populations]
**Concepts (9%):** The training and treatment concepts are congruent with the culture and values of the trainees as well as the values and culture of Black and Latine families.	*“Especially when it came down to do the intake session, and you ask them about like discipline and spanking and culturally to us, It's not* *…* *you hear Black parents saying, “Oh, yeah*”*, for us that's not alarming for somebody else, It might be alarming, or I'm not a parent that you* *…* *’ You kind of know they do, but they don't want to admit it out loud, because they don't want you to call people [DEPARTMENT OF HUMAN SERVICES] on them*”*.* [Barrier-Concepts-Poor Cultural Sensitivity to Physical Discipline] “*I see the benefits for the community that I work with and that I belong to. I see the benefits in helping parents create a relationship with their child. And, I think of a future where, within our community we see a lot of young moms, you know… and a lot of that has to do with that relationship that's never built, and then kids could make it to teenagers, and, you know, then they end up getting pregnant, or, you know, leaving home early because they don't have that relationship. So, to find that you could work* *…* *before the issues happen and build that bond between a mom and a daughter, mom and a son, whether they're Hispanic or African Americans, or of any ethnic background, makes it quite inviting.I guess as you project into the future, it makes this training quite significant for our community*”*.* [Strength-Concepts-PCIT is Valuable in Cultural Community]. “*The manual was great, because it's a specific, you know, it tells you exactly what you could say, how you could say, even though we always add our own words, you know, but it still give[s] us kind of like the structure of how to get it started. Or if we get confused, I'm just able to go back to the book and see where I should go. So, it was awesome.”* [Strengths-Concepts-PCIT Structure]
**Goals (22%):** The goals of the training align with the trainee goals; framing goals to mirror the values and needs of the trainees and clients.	*“Culturally speaking, I think some things were new for me* *…* *I think that lack of knowledge that I didn't even know I had when it came to other cultures* *…* *”* [Strengths-Goals-Black and Latine Competency Improved-Clinician Cultural Humility] “*What was memorable?* *…* *I think it was just kind of the sense of community that the hosts were able to kind of foster among us. It definitely felt like, even though we were all* *…* *in different agencies* *…* *it definitely felt like* *…* *there was a familiarity and a sense of like comfort that was fostered where we were able to speak freely about our concerns… I think* *…* *to have been across agencies and to still feel that connected, was pretty unique*”*.* [Strengths-Goals-Creating Community]. “*Yeah, so I currently still implement PCIT* *…* *I mean, PCIT therapy. I feel competent in it. And I think that came from the consultation calls and then getting that support*”. [Strengths-Goals-PCIT Competency Improved]
**Methods (15%):** Culturally adapted and non-adapted practices included in the training and intervention to help trainees and clients reach their goals.	*“I think it was the variety that it offered up. So it was the having the host family. It was* *…* *also having the time set aside for us to go over the materials and put them in practice. The visual aids and the videos helped as well, I think with the variety*”. [Strengths-Methods-Helpful Resources] “*I love the training* *…* *it was straight to the point and hands on. So to me that was great* *…* *so I loved it.”* [Strengths-Methods-Hands on Training, Role Play, Breakouts] “*I think the consultation calls to me, were a good strength. It allowed us to be doing the practice of PCIT, but also* *…* *get feedback in real time. And even though it's only once a week, it's great, because a lot of times* *…* *when they're like some self-paced and stuff, you may not be able to ask someone who actually implemented it questions, and we were given the opportunity to do that*”. [Strengths-Methods-Consultation Calls]
**Context (29%):** Consideration of contextual factors (e.g., acculturative stress, clinician burnout, social events, economic conditions) that impact the trainees and the clients they are serving.	*“It was a nice space to be able to go there with, you know, white clinicians present and like respectful and allowing space. So, I think that was like a refreshing experience, because, yeah, sometimes it's just not the case. So I feel like kind of going back to that cultural humility piece where that wasn't having to deal with like I don't know how to put it, but like accommodating like white discomfort, or anything like that, I just feel like the white clinicians that were there like made room for things and didn't make it about themselves* *…* *which is refreshing because they allowed [THE AFRO-LATINA LEAD CLINICIAN] to* *…* *lead* *…* *Even though they were in a position of authority they were able to just be present and attending like everybody else* *.* *and I feel like I’m used to having to code switch* *…* *I already kind of come in ready for that possibility. But it was refreshing that I didn’t have to like code switch, you know, because of the white presence being there. So, I think that was like refreshing for sure. It still doesn’t change the system that were in. So that's why I’m like* *…* *man, it was a nice space, but I didn’t leave there feeling like, “Oh, every other space moving forward is about to be like this* *…* *’ I don’t know, you know?*” [Strengths-Context-Training Climate] “*I felt like I was being acknowledged, understood rather than triggered* *…* ” [Strengths-Context- Validation and Healing of Racial Trauma- Felt validated, understood, & compassion vs. dismissed] “*Getting the patients in. That was really difficult for me. It took me a while to get people to kinda like trust or kinda come in into the program or just start the treatment*”. [Barriers-Context-Low to No Caseload & Client Buy-In & Engagement]

### Context

*Context* dimensional themes emerged as the primary barrier for clinicians, as well as a notable strength of the training. Several strength-centered contextual components were highlighted. First, most of the trainees indicated that the training climate was culturally affirming, that they appreciated the cultural humility shown by the white trainers involved in the training, and that it was a comfortable and safe space. One Black trainee indicated that she often would go to these types of training spaces expecting to have to code switch and accommodate white discomfort but was refreshed by the comfortability and space fostered during the training, where she could be unapologetically herself:

It was a nice space to be able to go there with … White clinicians present and like respectful and allowing space. So, I think that was like a refreshing experience, because … sometimes it's just not the case … I feel like, kind of going back to that cultural humility piece, where … not having to deal with like, I don't know how to put it, but like accommodating … White discomfort, or anything like that, I just feel like the white clinicians that were there like made room for things, and didn't make it about themselves … which is refreshing because they allowed [THE BLACK LEAD CLINICIAN] to … lead … Even though they were in a position of authority they were able to just be present … attending like everybody else … and I feel like I'm used to having to code switch … I already kind of come in ready for that possibility. But it was refreshing that I didn't have to … code switch … because of the White presence being there … that was … refreshing, for sure. It still doesn't change the system that we're in. So that's why I'm like … I mean, it was a nice space, but I didn't leave there feeling like, “Oh, every other space moving forward is about to be like this”.

Another theme centered around the validation and healing of racial discrimination trauma experienced by Black and Latine families. Trainees identified that they felt compassion, validated, and understood (rather than dismissed). For instance, one person stated that they “*felt like I was being acknowledged, understood rather than triggered*…” Although most of the trainees indicated that they weren't triggered in a negative way during the course of the training year, some trainees reflected that there were points of the training where they were triggered to get in touch with some of the racial discrimination events they had personally experienced in their daily lives:

 …You know, I've experienced discrimination and of course it's very hurtful. Actually … not too long ago I was at a store where the lady asked me to leave. She followed me around, and when I asked her, I was in there with my kids, “why are you following me?” She said, “well, if you don't like what I'm doing, just leave my store”.

Some trainees also reacted positively to the time devoted in the training to process national news events that were discriminatory and occurred during the training year (e.g., Buffalo Shooting). Further Contextual related strengths that were identified included the affordability, flexibility, and feasibility of the training. One trainee also highlighted that they appreciated how the trainers were able to tailor the training to their agency's specific needs and that the training greatly impacted not just their own cultural competency, but the cultural climate of [*redacted for review*] and their agency as a whole.

Among the contextual barriers identified, the trainees expressed that finding a common time everyone could meet was a challenge. Further, they stated that the training was time demanding—meaning that the 5-day training was too long for them, that they had difficulties getting their competencies done in 1 year, or that the 9:00am to 5:00pm structure of the training days interfered with their work schedule:

The only challenges had to do with more of just the time. I understand that it's 5 days, but that, that was a bit of a barrier challenge with me since I also work full time at a couple of different jobs. But aside from that, everything went well.

Next, some clinicians also described that meeting and increasing their competencies and feeling fully confident in their ability to deliver PCIT was a challenge because they did not complete the training or had too few PCIT clients, including autistic clients. Out of the trainees interviewed, only four were able to build caseloads that included autistic clients, two of whom worked at an agency that specialized in offering treatment services to autistic populations. One trainee expressed some hesitancy about their perceived competency because they were not able to have access to any cases during the training period. Lastly, completing the training and getting cases were challenges for some clinicians because they had to transition jobs or had challenges with client engagement.

Getting the patients in. That was really difficult for me. It took me a while to get people to kinda like trust or kinda come into the program or just start the treatment.

### Goals

Across all of the EVM framework elements, *Goals* emerged as the second most endorsed theme. The primary goals of the *Creating Community Initiative* were for Black and Latine trainees in [*redacted for review*] to improve their competencies in (a) delivering PCIT to (b) Black and Latine families and (c) autistic families. Thereby potentially increasing PCIT dissemination to Black and Latine families with autistic youth. All of the clinicians expressed that they felt more competent in their ability to deliver PCIT, specifically feeling more skilled in their ability to tailor PCIT to Black and Latine families. Half of the clinicians expressed feeling like they had greater cultural humility as a provider, while others felt more prepared in their ability to understand racial discrimination and how it impacted the families they served. One Latina trainee described having greater cultural awareness after participating in the training, a skill important to cultural humility development:

“…Culturally speaking, I think some things were new for me … I think, that lack of knowledge that I didn't even know I had when it came to other cultures…”

A majority of clinicians also reported their competency in delivering PCIT improved after the training. Some clinicians attributed this improvement to working with a variety of cases after receiving the training or getting consultation from the trainers. Others who had lower caseloads attributed this improvement to their practice of PCIT skills in other contexts (e.g., with their own children). Although most of the clinicians felt confident in their ability to implement PCIT, there were a few clinicians who were ambivalent in their ability to adequately deliver PCIT with their families—primarily because they were unable to have adequate practice with clients during the training. On top of developing their cultural and PCIT competencies, trainees also indicated feeling more knowledgeable about autism and confident in delivering PCIT with autistic children due to a variety of tools provided in the training (e.g., autism case examples, autism lectures, consultation call case discussion). For example, during a consultation call, a clinician sought feedback regarding how to support the Black mother of an autistic child who was having challenges engaging in play with their child because of the repetitive nature of the play. Another trainee remarked on the very positive experience of seeing a parent overjoyed after only one session, hearing her autistic child speak and being engaged with the play.

Outside of the goals embedded within the training, clinicians also identified their own goals related to the training that motivated them to participate. For example, some clinicians mentioned wanting to gain skills that would help them better serve Black and Latine communities and have greater flexibility in their ability to provide beneficial interventions to diverse families. Lastly, trainees indicated that they were greatly impacted by the sense of community built within the training, by the connections to other clinicians in different agencies, and by having a space with other culturally similar clinicians. Trainees described the goal of creating this community as unique, memorable, and motivating:

What was memorable? … I think it was just kind of the sense of community that the hosts were able to kind of foster among us. It definitely felt like, even though we were all … in different agencies … there was a familiarity and a sense of like comfort that was fostered where we were able to speak freely about our concerns … I think … for it to have been across agencies and to still feel that connected, was pretty unique.

While most described having greater cultural competency with respect to working with Black and Latine families, there were a select few who indicated that they felt no change in their cultural competencies because of their shared racial-ethnic background or previous experiences working with Black and Latine populations. Additionally, those whose confidence did not change regarding delivering PCIT to autistic populations attributed this to either (a) their lack of access to autistic cases during the training or (b) their extensive previous experience working with autistic individuals (e.g., Board Certified Behavioral Analysts).

## Method

A variety of *Methods* used during the training were well-received. In particular, trainees identified the following as beneficial: (a) resources; (b) hands-on training, role plays, and breakouts; (c) consultation calls; (d) practice with a volunteer Black family; (e) accessibility of the training; and (f) the training organization. Some resources trainees found helpful included the use of Canvas, video examples of Black and Latine families and with autistic youth, the demonstration of measurement tools (e.g., Eyberg Child Behavior Inventory), direct practice with the PCIT Manual, and the handouts provided:

I think it was the variety that it offered … It was the having the host family. It was … also having the time set aside for us to go over the materials and put them in practice. The visual aids and the videos helped as well I think with the variety.

For those who participated in the consultation calls, they highlighted that the consultations were vital in improving their competencies because it allowed them to get individual feedback and support for the cases they had in real-time.

Clinicians also commented on the digital training platform. For example, one clinician expressed challenges with navigating the online platform; however, most of the providers liked that the training was accessible due to the digital format. Among the recommendations provided by trainees, suggestions for the *Methods* were one of the most discussed dimensions. A select few stated that having more practice with the volunteer family, more specific feedback on their fidelity videos, and access to more video examples would have been helpful. There were mixed opinions with respect to the length of the training. Some advised that the training be condensed, while others suggested that the training be extended so as to do a more in-depth digestion of the training content. Thus, a couple of trainees recommended that trainers provide a follow-up training after the training year was complete.

### Persons

Themes stemming from EVM's *Persons* dimension arose as one of the more prominent themes within the strengths about the training. Within this broad category, two subthemes were identified. First, the trainees described various attributes about the trainers that contributed to their increased training satisfaction and competency (e.g., culturally diverse trainers, knowledgeable trainers). For example, they indicated that they enjoyed working with a culturally diverse set of trainers and that it helped contribute to their comfortability, cultural humility, and knowledge acquired throughout the training. For instance, one person stated:

I think it's good to have … individuals from different cultures to implement the training because they may bring different perspectives that we may not be aware of. So, especially with cultural humility, you have to keep yourself in check and keep questioning yourself about other people['s] culture. So, if you're exposed to those cultures, you can have a general idea of how to be more culturally competent.

In addition to having a culturally diverse set of trainers, trainers were further described as good, knowledgeable, and experienced with respect to delivering PCIT, delivering PCIT in a culturally sensitive manner, and working with autistic populations. One Black trainee reflected that having an Afro-Latina clinician lead the training and observing the collaboration and connection between the Afro-Latina trainer and the white trainers created a sense of allyship and safety. When obstacles did arise, trainees stated that they felt that the trainers were responsive to their needs and demonstrated good problem solving. Trainees also indicated that trainers were good at explaining concepts and skills, they appreciated the individual feedback given by the trainers, and they enjoyed the PCIT parallel process utilized. For instance, one trainee commented on the trainers' use of labeled praises to provide feedback: “*One thing that they did…was telling us we’re doing a good job*”.

The second most prominent theme that arose within the *Persons* EVM dimension centered around the racial-matching embedded within the training. Trainees expressed that the racially-matched training created a community for them, impacted their motivation and engagement to enroll and continue the training, facilitated cultural tailoring, and allowed them to better understand their clients' experiences because of their “*inside perspective*”. For example, one Black female trainee alluded to the fact that the racial matching with the trainer and herself helped her feel more confident in her ability to deliver PCIT:

Having a trainer … also from a similar background … made me feel like I could do it vs. being like, “I don't know about this”, but to hear that it could work, and it does work. So that encouraged me to implement PCIT.

Others expressed that the racial matching from the community built within the training cohort was inviting, prompted their interest in the training, and positively impacted their experience:

I work with my Hispanic community and the African American community … We only see each other at big meetings when we go to [CONFERENCE NAME]. So, having a small group of clinicians that are mainly Hispanic and African Americans, it made it quite significant … When I read the title, [to]make it a community, you know … culturally competent, it made it quite inviting and interesting for me.

Although the majority of the trainees voiced that the racial-matching within the training positively impacted their motivation and engagement to enroll and continue the training, a few of the clinicians noted that the racial matching did not influence them, rather they were influenced by the quality of the persons providing the training and learning a new intervention to support Black and Latine families.

Clinicians in training also provided some person-centered recommendations to improve future trainings. Specifically, one Black male trainee suggested expanding the diverse cohort of trainers to include male PCIT trainers. In addition, another trainee recommended that it would be helpful to include a volunteer family in the training that had an autistic child to further their skill practice with autistic populations.

### Concepts

Within the *Concepts* EVM dimension, trainees made note of some issues with the discipline concepts of PCIT that negatively impacted their ability to deliver PCIT in a culturally responsive and flexible manner. Some clinicians asserted that they felt like the *timeout* procedure was not appropriate for the racially minoritized individuals they served, while others expressed some reluctancy to broach the conversation of discipline and spanking with Black families:

…When it came down to do the intake session, and you ask them about like discipline and spanking and culturally to us … you hear Black parents saying, “Oh, yeah, for us that's not alarming”, but for somebody else, it might be alarming, or you kind of know they do, but they don't want to admit it out loud, because they don't want you to call the people [DEPARTMENT OF HUMAN SERVICES] on them.

Although there were some issues brought up with the way the PCIT manual discussed discipline, clinicians also outlined strengths of PCIT's *Concepts* that led them to view PCIT as an acceptable intervention for their clients. Some clinicians provided feedback that they viewed PCIT as a valuable intervention for the Black and Latine clients in their community and agency as well as the autistic population. For example, clinicians specified that within the cultural community, PCIT is valuable because it has the capacity to build family bonds and improve overall family functioning:

…I see the benefits for the community that I work with and that I belong to. I see the benefits in helping parents create a relationship with their child. And, I think of a future where, within our community we see a lot of young moms, you know, and a lot of that has to do with that relationship that's never built, and then kids could make it to teenagers, and, you know, then they end up getting pregnant, or, you know, leaving home early because they don't have that relationship. So, to find that you could work before the issues happen and build that bond between a mom and a daughter, mom and a son, whether they're Hispanic or African Americans, or of any ethnic background, makes it quite inviting … I guess as you project into the future, it makes this training quite significant for our community.

Further, trainees denoted that for caregivers with autistic children, PCIT provided hope and evidenced-based skills so that caregivers could better engage and interact with their autistic child. One clinician shared:

A lot of times there's this misconception that autism is something … that doesn't have the support that it needs to be able to encourage the family to strive. So, the fact there were research-based information on how families were assisted … by PCIT and seeing that change of … how the child used to be before PCIT and seeing the level of engagement … and it actually being helpful, was really great…

When asked about what aspects of the training were triggering in respect to discrimination trauma, some clinicians pointed out that because PCIT was created with a white framework and with white families, implementing the intervention with Black and Latine families was triggering. Similarly, another trainee who had some background in applied behavioral analysis (ABA)—a commonly delivered, behavioral method for skill-building in autistic youth—found challenges with the timeout protocol in PCIT primarily due to the divergence from their own professional and training experiences, saying they were off-put by the *timeout* procedure because, to them, it mimicked a “*jail cell*”. Although some clinicians expressed some cultural concerns with PCIT as a treatment, others appreciated that PCIT was a structured, positive intervention that provided parents with clear skills. Further, they recognized it as an evidenced-based treatment benefitting many families across numerous research trials.

### Content

Trainees highlighted *Content*-related adaptations within the EVM framework that strengthened their experience of the training. For instance, they described enjoying the portions of the training that enabled them to learn how to implement and tailor PCIT to autistic children, and Black and Latine families. In particular, one clinician reflected on the discussion topics held during the training surrounding racial discrimination experienced by Black families, and how that impacted their delivery of PCIT to Black families:

So, we had a day where we kind of talked about discrimination in the Black community and … our experiences in our own cultures and with the world. And one of the [Afro-Latina lead] trainers, she described the time where her son was actually like stopped by the police and how … it was, you know, very scary for her … her Black son being stopped by a cop, and she luckily was there … able to … intercept the situation. But we … know nowadays, sometimes the parents are not there, and they [the police] don't care. So, it was really nice to be able to kinda still be trying to talk about PCIT, but ultimately kind of talking about the elephant in a room. Where we may be giving our parents this therapy, giving them this training for the better, the family and stuff, but there is still these outside forces in the world who… don't care.

Another clinician also discussed how the training was a benefit to their agency and the families they served because it not only enhanced their competency in delivering a new EBT, but was tailored to increasing their understanding of how to deliver the treatment to Black and Brown autistic families:

“The agency in which we work for services children with autism and typically our clients are Black and Brown. So, this was a great addition to some of the services we already provide”.

### Language

Throughout the interviews, themes related to *Language* made up a minority of the overall themes and were emphasized primarily by the Latine trainees who made up a smaller percentage of the training cohort (*n* = 3). The trainees who identified as Latina provided positive feedback about the training's adaptations (e.g., translated materials) that allowed them to deliver PCIT in a linguistically concordant manner. Further, both Latina clinicians expressed that having the resources allowed them to deliver PCIT more easily in Spanish to their clients who were less proficient in English. One Latina trainee even noted that for some of her families, having the ability to receive services in Spanish allowed her clients to feel more comfortable speaking up and feeling understood.

While the majority of the Latine clinicians provided positive feedback about the language level adaptations included in the training, some of the Black trainees expressed that the language embedded within the standard (non-adapted) PCIT protocol was culturally “offensive” to Black families and that it required extra work on the part of the clinician to tailor the intervention to be ethnically-racially sensitive. One clinician even hinted that some of the language used in the protocol, specifically when it came to discussing discipline, was accusatory and assumed families were harming their child.

The most challenging, I would say, is the actual protocol itself. I would say a lot of the words in it aren't very culturally inclusive for everyone. So sometimes we found ourselves kind of like trying to find a different word that would fit, you know, our community, because sometimes you know, some words can be offensive to some families, because they may try and say like, “Ooh are you trying to say that I'm probably harming my child or something?”

## Discussion

Investigating clinician perceptions of training satisfaction and EBT acceptability following training is a vital step in determining if an EBT will be adopted in a clinician's practice (39, 40). But implementation research has largely failed to explore the impact of EBT trainings for racially and ethnically diverse clinicians especially those serving neurodiverse or racially and ethnically diverse communities (1, 29, 31, 32). The present pilot study explored perceptions of a culturally adapted PCIT training for Black and Latine clinicians serving autistic youth in (*redacted for review*) using an EVM framework. Our findings illuminated EVM elements of PCIT training that impacted clinician training satisfaction and engagement, as well as EBT skill acquisition and acceptability. The results also provide insight into training areas that could benefit from improvement before disseminating more broadly to additional training cohorts.

### Training elements which improved clinician competencies: persons, context, goals, method, concepts, & language

#### Persons & context

Consistent with EVM's *Persons* dimension, clinicians identified trainers’ attributes as being *knowledgeable experts* who were *collaborative* and *culturally diverse* as salient to their improved competencies. Black and Latine clinicians perceived the trainers as having expertise in (a) in delivering PCIT to racially and ethnically diverse groups and autistic populations and (b) their ability to train mental health providers to comfortably do the same. Such expertise was also considered a valuable mechanism for increasing PCIT's reach and accessibility among the Black and Latine communities by increasing the number of providers of color who were comfortably equipped with these specialized PCIT skills in serving their Black and Latine communities (41).

Although prior PCIT dissemination efforts utilized trainers with considerable expertise in PCIT, the importance of *collaboration* within training cohorts, building *cultural competencies*, and targeting training cohorts from diverse backgrounds to enhance dissemination efforts has not been as widely recognized (42, 43). Clinicians remarked on the visible collaboration among the diverse cultural identities of the trainers in the *Creating Communities Initiative,* which appeared to facilitate a sense of trust between the White trainers and the Black and Latine trainees. Such modeled collaboration among the trainers paralleled similar processes strived for in cross-cultural therapy (44).

Specifically, the racially/ethnically diverse training team and matched trainee cohort was a unique component and strength of the training. Some trainees reported that they were accustomed to being in largely white training spaces, and as such the diverse climate created by the *Creating Community Initiative* was refreshing. Participating in a training with other clinicians with similar racial/ethnic backgrounds created a community in which trainees felt more motivated and engaged. This is a critical insight as it suggests that providing therapists of color with safe spaces with other racially/ethnically similar providers may increase trainee's buy-in with EBT's, that they may otherwise distance themselves from because of their Eurocentric origins. Mood and Sandage (45) in their citing of Traister (46), announced:

“People of color have been taught to take in injustices, microaggressions, and racism, and hold in the anger because one will likely be punished for expressing that anger and then expected to fix the mistake” (46).

Thus, the findings of the current pilot revealed that the racial-ethnic matching incorporated in the PCIT training program was well received. Further, Buche et al. (47) suggests that such diverse and racially/ethnically matched training teams who share an understanding of the lived experiences of their trainees, and the created supportive communities of ethnically/racially matched clinician cohorts, offer protective buffers against discrimination stress and burnout. In fact, Mood and Sandage (45) go on to state that such racially diverse training settings in which persons of color are permitted to be in the foreground, while their white counterparts, who are systemically viewed to hold power, occupy space in the background, offer added benefits for catharsis and healing.

“Groups such as these allow for therapists of color to express anger, frustration, and fatigue without being misunderstood or dismissed by White colleagues or expected to take care of the feelings of White colleagues” (p. 8).

This sentiment was further illuminated in the current pilot by EVM *Context* dimensional themes, in which Black and Latine clinicians described the training climate as “*comfortable*”, “*safe*”, and “*culturally affirming*”. One trainee noted the cultural humility of the white trainers, and how it fostered a comfortable environment where she didn't have to code-switch, nor accommodate for white discomfort. This is significant because it points to the strain “white spaces” can have on clinicians of color and demonstrates the benefits that can result when white professionals in the spirit of allyship, intentionally refrain from taking a stance of superiority. Due to the dominance of white spaces, Black clinicians, much like our referenced trainee, feel pressured to codeswitch or conform to white norms in an effort to remain “professional”, perpetuating tension, discomfort, and rejection of Blackness (28, 48, 49). Given the salience of whiteness that pervades most organizational and societal realms—including PCIT (17)—it behooves EBT trainers to consider how to create safe training spaces for Black and Latine clinicians' voices to be heard and valued, towards seeking the expansion of EBTs, and making them culturally applicable to communities of color while also not overburdening the limited number of minority trainers to do this work alone.

#### Goals & methods

Clinicians seemed to confirm that the EVM *Goal* of increasing Black and Latine clinicians' competencies in serving Black and Latine families and autistic populations and PCIT delivery were met. Regarding the goal of increased competency in working with Black and Latine families, several trainees expressed developing greater cultural humility (e.g., self-awareness) and feeling more skilled in their ability to culturally tailor PCIT as needed (e.g., discussing racial discrimination in Black families). The majority of clinicians also noted increased competency in their ability to work with autistic populations due to various methodological components of the training (e.g., autism case discussions, autism lectures during consultation calls). These are exciting and promising findings, as families of color call for more culturally humble clinicians as well as racially/ethnically diverse clinicians who are knowledgeable about providing care to autistic youth (6).

In addition to cultural and autistic competency, most trainees described feeling more competent in their ability to deliver PCIT, much of which they connected to a variety of EVM *Methodologies* embedded throughout the training (e.g., consultation calls in which specific Black and Latine cases, autistic youth cases; breakout rooms for skill practice; video demonstrations with Black and Latine families, autistic children; use of a volunteer Black family for the training). With respect to the consultation calls, such findings make sense given that previous implementation and dissemination research suggests that consultation call attendance improves clinician skill development and treatment adherence (42, 50).

#### Concepts

After partaking in the training, many trainees viewed PCIT as a valuable intervention for Black and Latine families, autistic youth, and their agencies. They appreciated the structured and evidenced-based nature of the intervention, its capacity to improve engagement for families of autistic youth, and its ability to strengthen bonds within their cultural community. Studies highlighting the perspectives of Black and Latine families of autistic children have illustrated the stigma, fear, and stress communicated by Black and Latine caregivers, which may negatively impact the parent-child relationship (6, 51). Black and Latine clinicians in this study either witnessed or recognized the ability of PCIT to increase positive interactions between Black and Latine caregivers and their children, which has also been mirrored in a multitude of research and clinical endeavors (52). Finally, most of the trainees (75%) expressed that they would like to either continue training in PCIT or continue implementing PCIT with their Black and Latine clients. This lends support for the high acceptability of the intervention [87%; (53)] and illustrates the small net that has been casted to expand PCIT dissemination to Black and Latine families in (*redacted for review*).

#### Language

The EVM *language* dimension made up a minority (2%) of the themes endorsed by the participating clinicians, likely due to the lower sample size of Latine clinician trainees (*n* = 3) relative to that of the Black trainees (*n* = 5). Thus, when there was a 100% endorsement by the Latine trainees on issues of language, that is noteworthy. With that said, a few training elements emerged that trainees reported as positively impacting their competency. For example, all of the Spanish-speaking Latine trainees found that having access to PCIT materials in Spanish enabled them to provide services to their Spanish-speaking clients and made the families feel more comfortable in PCIT sessions. Likewise, other studies have echoed that Spanish-speaking families may feel uncomfortable interacting with personnel and professionals due to language discordance (54). Progressively, advancements in this domain to provide adapted versions of PCIT to Spanish-speaking families have shown moderate success in reducing child disruptive behaviors and improving retention rates (1, 55).

Although there have been efforts to test culturally adapted versions of PCIT for Latine families, less clinical attention has been paid toward training Spanish-speaking clinicians in PCIT, especially for autistic individuals. This is an important adaptation as the large-scale PCIT dissemination projects have been made up of mostly white female clinicians working with neurotypical children (53). A recent study that examined the role of race and ethnicity in clinician experience delivering EBTs found that Latine clinicians made more augmenting adaptations (e.g., language adaptations) and reported fewer client disengaged behaviors than White therapists (56). Likewise, language was also noted as the primary adaptation used among a set of clinicians delivering PCIT to Black, White, and Latine populations (57, 58). The positive response in the current pilot by 100% of the Latine clinicians suggests that trainees may benefit from receiving translated materials to seamlessly provide services in Spanish for their Spanish-speaking clientele, which creates a more comfortable, culturally affirming environment. However, further language tailoring is recommended as translated materials may not adequately represent the diversity within Spanish-speaking Latine families (58, 59). Accordingly, it is noteworthy that PCIT-International Association is currently undertaking improved translations of their Spanish materials.

### Barriers limiting clinician competency and recommendations for future trainings: context, language & concepts, goals, & persons

Trainees described barriers they experienced during the training related to *Language, Concepts, Goals*, and *Context* dimensions. These insights shed light on areas of the training that could be improved upon, as well as potential limitations of PCIT as a treatment for Black, Latine, and/or autistic families in its standard protocol (i.e., without adaptations).

#### Context

*Contextual* factors such as time demands and caseload establishment served as the most impactful training barriers. For some, meeting the time demands of the training, while trainees also balancing their other agency work responsibilities, was challenging. The length of the training was comparable to other PCIT training models, yet other researchers have similarly proposed limited accessibility for the current EBT training structure [e.g., week-long 40 h training days, 2-day advance training, year-long bi-weekly consultation calls; (32, 33, 42)]. As a result, if replicated, participants from the current study recommended that the training be condensed to a shorter time interval. While EBT trainings have remained consistent in their structure for many years now, some attempts to shift structure based on clinician feedback have been attempted, albeit with minimal success (32, 33, 60). Indeed, one such study built an EBT training around clinicians' desires for fitting the training into existing agency practices, and for the training to occur over a longer period of time [i.e., 8 h training days were broken up into 21-weekly 90 min to 4 h sessions over the course of 6 months; (32, 33)]. Ultimately, minimal improvements were found across observations of clinicians' skill use, thus, raising the question if accommodations are effective (even if seemingly more desirable).

Next, some clinicians in the present pilot noted difficulty with building their caseload (75%) and initiating client buy-in (25%) during the training year. However, such challenges are not uncommon in community-based implementation initiatives where agencies or clinicians report trouble building caseloads, agency instability, and/or getting connected with referral sources to implement EBTs (59, 61). Implementation researchers have suggested the importance of agency buy-in (62). With respect to the current pilot, while some agencies provided a great deal of support (e.g., administrators attending both the 5-day and 2-day trainings, consult calls, pursuing their own certification), other agencies did not. In the case of unsupportive agencies, trainees experienced a paucity of referrals, and staff turnovers were also witnessed to negatively impact trainee certification. Thus, future efforts should build caseload capacity prior to training to ensure clinicians can start immediately seeing clients and practicing the EBT.

#### Language & concepts

Some of the Black trainees drew attention to the cultural and professional mismatch between the *language* used in the PCIT protocol, the timeout procedure, and the experience of Black families. Black trainees deemed some of the language in the treatment manual as “*offensive*”, especially regarding the language used when discussing discipline with families (e.g., timeout). Clinicians feared that the language used in the standard protocol might lead Black families to believe that they were being accused of harming their children (i.e., spanking). Hence, clinicians noted that they often had to adapt the language on their own to make it more culturally appropriate. Within Black communities, language is a prominent device typically used to empower and uplift one another. According to some of the clinicians, language was less applicable when families were trying to meet goal criteria in CDI (63); however, language was an issue when administering the PDI protocol of PCIT (64). Moreover, similar language adaptations have been pointed out in other studies centered on the experience of Black and Latine families and clinicians (57, 58).

Although concerns regarding the language used in PCIT surrounding discipline with Black families has yet to be raised in the literature, there seems to be a general agreement among researchers and clinicians that spanking is not recommended (65). Spanking may be a social norm for some families and may be wrongly associated with corporal punishment, thus, broaching the topic must be done in a culturally sensitive, empowering, collaborative, and non-judgmental manner. Findings from the present study suggest that more in-depth conversations and practice on how to talk about discipline with Black families (in ways that promote unity between the clinician and family) is needed. Psycho-educational videos, educational handouts, discussion about caregiver childhood experiences, and parenting assessment tools have been noted as alternative ways to initiate the conversation of discipline with caregivers (35, 66, 67). It should be noted that the trainers in the current study only altered the training model with recommended considerations for DPICS coding and African American Vernacular English (AAVE), but it did not alter the PCIT manual in any way. Thus, future PCIT studies may benefit from exploring language used in the PCIT manual itself to enhance cultural consciousness (35, 63).

#### Goals

Despite challenges with building their caseload and eliciting client buy-in to PCIT, all participants indicated feeling increased competency in at least one of the *goal* areas of the training (i.e., Black and Latine cultural competency, autistic competency, PCIT competency). Yet, a few trainees did cite ambivalence or no competency improvement in some domains. For some, PCIT or autism competency development remained a challenge because of their minimal caseload and lower exposure to cases to practice the skills. Because of this barrier, trainees recommended having a follow-up training after the consultation year to allow more time to dive deeper into some topics, continue to practice, and maintain the supportive community of professionals of color.

For others, they endorsed no cultural competency improvement because of their inside knowledge from being members of the Black and Latine communities and previous competency stemming from clinical training or treatment delivery. For example, Black therapists voiced that they were better able to understand the unique experiences of their Black clients, improving the care they provide (57, 68, 69). Given the complexity of understanding and practicing cultural humility, the skills demonstrated by clinicians of color to address the needs of their diverse clients has been historically undervalued (45). For instance, when practicing cultural humility, therapists of color may have to navigate potential counter transference related to racial trauma (45). Consistent with the EVM *Context* of the culturally adapted PCIT training and acknowledged by the Black and Latine clinicians, participants indicated the importance of taking time during the PCIT training to process systemic racism and discrimination at multiple levels: (a) as trainees, (b) in their transitional roles to clinicians/disseminators of PCIT, and (c) at the level of their Black and Latine clientele's lived experiences receiving PCIT within such contexts. Accordingly, Moon & Sandage (45) highlight the importance of implementing support for clinicians of color who are seeking to practice cultural humility, especially in the form of organizational and institutional supports, as the perspectives of clinicians of color may not be taken into account when promoting the practice of cultural humility.

“It is important to recognize that the constructive practice of virtues like cultural humility requires highly differentiated and diversity-sensitive communal and organizational contexts to support the individual clinicians seeking to practice cultural humility”. (p. 7)

#### Persons

Although the racial and ethnic diversity of the training was a notable strength, trainees provided some suggestions about gender and neurodivergence to expand the diversity of the training experience, such as the inclusion of male trainers. Unfortunately, over the past decade, the number of male clinicians in mental health has substantially declined and is projected to continue to shrink (70, 71). The preclusion of male trainers as well as autistic trainers and trainees in clinical spaces is a problem because it undermines the role of the intersection of race, ethnicity, neurodiversity, and gender on therapists' experiences (47). In fact, studies have found that clinician characteristics such as gender may influence male clinician's use of EBTs (72, 73). Indeed, in this pilot, all of the male therapists (*n* = 4) who initiated the training did not go on to successfully graduate cases throughout the training year. Secondly, another trainee suggested that the learning experience could have greatly improved if the volunteer family (i.e., Black mother, neurotypical Black son) had been with an autistic child. This may have also been an important addition for trainees as some of them expressed difficulties finding autistic cases during the training year and practicing their PCIT skills to autistic populations.

### Limitations and future directions

Several limitations to this pilot study need to be acknowledged. First, the study included a small sample of master's level clinicians who served Black and Latine families in (*redacted for review*); thus, the findings from the study may not be generalizable to other Black and Latine clinicians who live in different communities (e.g., rural) and states. Further research should replicate the *Creating Communities Initiative* in other regions. Another important limitation was the absence of a comparison group. In the future, comparison samples of Black and Latine clinicians who receive a standard PCIT training should be compared to clinicians receiving the adapted training protocol to determine the value and difference the culturally grounded training provides.

The scope of the pilot was also limited to only clinician outcomes rather than also reporting on family-level outcomes. Although we intended to receive data on family behavioral outcomes, clinicians had challenges with engaging their clients in the research process during the training year. Currently, Black (7.7%) and Latine (9.4%) autistic individuals are highly underrepresented in the autism intervention literature, creating a gap in our knowledge of treatment efficacy for Black and Latine autistic youth (74, 75). Even though the research team was able to get clinician report data, to date, no research has been undertaken to explore Black and Latine family perceptions of PCIT and its applicability to their culture and needs. Given the difficulties with family engagement highlighted in this study, as well as previous studies, further research examining Black and Latine autistic family perceptions of PCIT using a community-participatory based research model would be impactful in guiding future PCIT adaptation and dissemination efforts.

Given that one of the primary goals of this initiative was to not only expand dissemination of PCIT to Black and Latine autistic families, but to deliver PCIT in a manner that is meeting their needs and values, the study could have benefited from including perspectives from autistic individuals, families, and professionals in the development process. Families have voiced wanting more collaboration between professionals and themselves so that they can be more involved in the development of programs and interventions being created for their children (6). We also did not obtain demographic information from the clinician regarding the neurodiversity of the trainees, an important addition to include in future studies to amplify perspectives from Black, Latine, and neurodiverse clinicians. Thus, if replicated, using a community based participatory research approach can enhance the voice of autistic individuals and families in this process to ensure that the training and intervention is being delivered and disseminated in a way that aligns with their needs and values, enhancing potentially treatment buy-in and treatment retention for these families.

Future efforts should consider ways to continue the community built in the *Creating Communities Initiative following training*. Although the training team encouraged continued communication between themselves and the clinicians for future consultation and support when needed, there are a few innovative ways this could be addressed. First, a professional mentorship model may be used to pair trainees with experienced PCIT clinicians of color to assist them with continued learning and support. Currently, this has not been adapted in a clinician training model. However, it has demonstrated success in supporting scholars and professionals of color (76). Second, a platform such as a list serve or continued use of the LMS course (e.g., Canvas) could be utilized to connect clinicians in the cohort and to field questions or provide ongoing consultation and/or support after the training year has concluded. PCIT International currently has a listserv for Certified PCIT Therapists and runs monthly collaborative consultation calls (pcit.org), but Black and Latine providers are vastly underrepresented in this space. Having a space developed specifically for Black and Latine clinicians to discuss EBT implementation or their questions may help the likelihood of PCIT implementation sustainability for historically underrepresented groups (47). Organizational and financial support are key for this type of effort, which may initially have added expenses (e.g., mentorship model or co-training). Our work is one of a dearth of studies that exist exploring the impact of culturally adapted training spaces. Additional empirical studies including randomized controlled trial of training involving clinicians of color are necessary steps to determine how best to optimize EBT trainings for clinicians of color. Financial support from both foundations and state and federal agencies is essential, but it depends on these organizations recognizing the value of developing culturally affirming mental health spaces.

### Clinical implications

At the heart of the present pilot findings, Black and Latine providers tell us that there is strong motivation and interest in the best practices that can serve their Black and Latine families of both autistic youth and non-autistic youth. Addedly, their desire is for such EBTs to offer a welcoming space in which feedback from both providers and families of color about enhancing EBTs (e.g., accessibility) can be fostered. Based on the current pilot findings, it is suggested that efforts focus on (1) increasing EBT training initiatives at an organizational level (e.g., incorporate cultural components into standard PCIT training models) and (2) shaping within agency support for providers of color to promote engagement and competency building in EBTs.

Incorporating cultural components into standard EBT training models (e.g., PCIT) at the organizational level should be an essential initiative for enhancing EBT dissemination and implementation efforts to Black and Latine communities. Specifically, PCIT International Association has taken some steps to identify a few persons of color to step into leadership roles to enhance communities serving racially and ethnically diverse communities. Yet, representation is only an initial step, and what is required next are welcoming, insightful discussions about how to continue the infrastructure that will increase the presence of Black and Latine clinicians and communities within the organization; the work is ever-evolving. When EBTs originate in oppressive systems, in the spirit of allyship, it behooves organizations and leaders to be extra mindful and purposely intentional of opening doors for increased accessibility and inclusion of Black and Latine providers. In restructuring such systems, key goals are as follows: (1) build pathways for successful EBT implementation, dissemination, and sustainability within Black, Latine, and autistic populations through reducing disparities in access in EBT trainings and increase inclusivity and (2) make such EBTs and trainings culturally attuned to the targeted populations of color, which will ultimately serve to increase behavioral health care for culturally minoritized families.

Secondly, agency support and adequate referrals to build caseloads are required to enhance clinicians' service within their community. Clinicians with more agency support (e.g., administrator training attendance/buy-in) are often more successful in completing EBT training and building skills (77). Support-building may also be required to combat barriers impacting Black and Latine clinicians [e.g., clinician burnout, high caseloads, racial discrimination; (28)]. For therapists of color, asking them to practice cultural humility without organizational and policy supports in place puts them at risk for increased burnout and turnover (45). Future trainings should include discussions and resources around how it is to be a Black or Latine clinician working in an agency where the clinician feels little power or support. This could inform the building of infrastructure to provide them with such supports, as was done with the secured foundational funding in the *Creating Community Initiative*. Through the provision of funding, some agencies were able to build out PCIT specialized rooms and given additional resources in families' language of preference (e.g., ECBIs and manuals in both Spanish and English). In addition, working with agency administrators to support Black and Latine clinicians through time off for initial training and biweekly consult calls may also increase agency buy-in to see firsthand the value of PCIT and training demands. To conclude, further efforts are necessary in increasing the visibility of the agency and the EBT through building an alliance with communities of color. Alliance formation between agencies, providers, and communities of color is essential to build trust within their neighborhood agencies, as well as the EBT to support the Black and Latine caseload needed for certification of their Black and Latine providers.

## Conclusion

This pilot offers preliminary support for the development of a culturally responsive PCIT training—organized under the EVM framework—for Black and Latine clinicians to build PCIT, cultural, and autism competencies. Our Black and Latine trainees inform us that creating a racially-ethnically matched community for Black and Latine clinicians promotes a safe, culturally rich, and motivating climate for clinicians to learn new EBTs to serve Black and Latine families of autistic youth. Time constraints, low caseloads, and poor agency support were identified as barriers to training completion and competency development. Considerably more work will need to be done to expand this initiative to increase the dissemination of PCIT to Black and Latine clinicians, families, and autistic youth.

## Data Availability

The raw data supporting the conclusions of this article will be made available by the authors, without undue reservation.

## Footnotes

^1^Quetsch LB, Chavez F, Onovbiona HU, Scudder AT. Creating communities: a pilot trial of a culturally adapted PCIT training with Black and Latine clinicians serving autistic youth (under review).
