# Supplementary material for: A qualitative analysis of a culturally adapted PCIT training for black and latine clinicians: creating communities for providers of autistic youth
Source: Front Child Adolesc Psychiatry. 2025 Jun 3;4:1517169. doi: 10.3389/frcha.2025.1517169 (PMC12170612; doi:10.3389/frcha.2025.1517169)
Supplement: Supplementary file 1 [file Table2.docx]

**APPENDIX A**

| *Training Evaluation Questions*: The following questions pertain to your evaluation and appraisal of the PCIT Training Experience.   1. What was the most memorable aspect of the training or training process (e.g., 5-day training, 2-day advanced training, consultation calls)? What will you take away from this training? 2. What were the strengths of the training? 3. What did you find most challenging about the training? Please indicate any barriers you experienced. 4. What are your thoughts on participating in a training conducted by trainers from different racial and ethnic backgrounds (i.e., Black lead trainer, white supportive trainers, graduate student Black and Latina trainers)? 5. What resources were most helpful during the training? Note. If further clarity is needed provide resource examples – start-up funding/resources, handouts, translated materials, video link, online course content, assessment measures. 6. Based on your training experience, do you feel competent enough to implement PCIT treatment? Why? 7. Were there aspects of the training that allowed you to feel more competent in working with Black or Latine children and families? What were they? 8. Were there aspects of the training that allowed you to feel more competent in working with families of autistic children? What were they? 9. What do you wish you had gotten more of or less of during the training? |
| --- |
| *Discrimination Trauma*: The following questions pertain to *Discrimination Trauma*, which is defined as the cumulative effects of racism/discrimination experienced either directly or vicariously. Discrimination trauma can be caused by one acute experience of racism/discrimination (e.g., sexual and racial harassment) or by numerous, more subtle forms of racism/discrimination that accumulate over time (also known as *microaggressions*). Racial Trauma has been linked to an individual’s mental and physical health.   1. As a Black/Latina/o clinician, were there elements of the training which you found triggering with respect to discrimination trauma? If so, what were they? And why? 2. As a Black/Latina/o clinician, were there elements of the training that you found healing with respect to discrimination trauma? If so, what were they? And why? 3. Given the effort to have a group of clinicians that were racially and culturally similar, how much did the offering of a racially/culturally matched cohort (e.g., advertised cohort specific to Black/Latino/a clinicians) impact your willingness to 1. Enroll, 2. see clients using PCIT, 3. continue in training, and 4. complete training competencies throughout the training year. 4. Do you feel like you had adequate support? (e.g., from providers, family, friends, community) |

APPENDIX B

**Cultural Training Adapted Curriculum Content Syllabus**

**(REDACTED FOR REVIEW)**

- *Conveying respect* through the salutary address of “*Ms*.” or “*Mr*.” even with the children.
- *Diversity of familial constellations* among Black and Latine Families: Discussion of *Kinship Supportive Networks* of both *Non-Fictive* and *Fictive Kin*, and making sure all the relevant parties are invited from the onset of treatment.
- *Taking It to the Streets*: PCIT clinicians and agencies creating community partnerships with culturally-sanctioned agencies (e.g. churches, community centers, afterschool Programs)
  - *Garnering PCIT support and Credibility*: Offsetting within-community scrutiny of using PCIT skills, which may appear antithetical to cultural norms.
- *Giving Credit where Credit is due*: Cultural considerations for using a highly language-based Eurocentric observational coding system (using the Dyadic Parent-Child Interaction Coding System (DPICS) Coding Manual, 2019) in assessing caregiver progress of target skills, when speaking Spanish with its multiple dialects, or for Black families who choose to use African American Vernacular English (AAVE).
- *Addressing the impact of Racial Trauma* on child behavioral symptomatology expressed as increased externalization and internalization behaviors
  - Initiate early cultural assessment of racial trauma and racial socialization practices
  - Processing daily lived experiences within their communities which may compound vicarious experiences of racial trauma, for improved comprehension of the family life context impacting their overall psychological functioning (Heard-Garris, Cale, Camaj, Hamati, & Dominguez, 2018).
    - Addressing neighborhood/world threats/current events contributing to vicarious trauma
  - Creating a safe holding space for processing and validation of such experiences for Black and Latine clinicians as windows of insight into their Black and Latine client’s experiences
  - Ensuring public safety in the management of children’s behavioral expression through both externalizing and internalizing symptomatology.
  - Managing public scrutiny from both members inside and outside their racial/ethnic communities for using PCIT, which may not be well understood, accepted, or appear antithetical to cultural norms.
- *Racial/Ethnic Socialization Parenting Practices & PCIT*
  - Using the PRIDE skills for managing anxieties around the common rite of passage that many caregivers of color have in engaging in the infamous “*Talk*" (Anderson, Caughy, & Owen, 2021) as part of racial/ethnic socialization parenting practices (Caughy & Owen, 2015; Kinouani, 2021).
  - Incorporation of culturally matched and affordable resources (e.g. handouts, toys, and game selections).
  - ethnically/racially matched toys and culture centered games fitting with PCIT tenants for interactional play in which the child and caregiver may relate to one another. – mechanisms for fostering cultural pride and healthy cultural identity.
  - Using PRIDE Skills to foster cultural racial ethnic identity pride and positive self-image and self-esteem
- *Making Time & Eat Your Wheaties*: Taking extra process time in treatment to address and problem solve when PCIT is not culturally resonating. Some examples are as follows:
  - Engaging in problem solving strategies for barriers to treatment by addressing Obstacles families face regarding treatment attendance (e.g. conflicts with work schedules and transportation issues) and compliance.
  - Difficulty being able to incorporate daily 5-minutes of special time play homework to practice the PCIT skills with their child due to other competing demands on their time (e.g. work schedule and meeting other children’s needs).
  - Offering additional supports for the caregivers and fostering healthy self-care strategies within the availability of their cultural context.
  - Incorporating additional preparation time for safe, careful, and thoughtful planning and prepping for implementing PCIT with the potential of a Timeout sequence for public misbehaviors, which may enlist the management of public scrutiny, and how to foster safety for themselves and their children from interactions with policing authorities
